# Automated detection and classification of concealed objects using infrared thermography and convolutional neural networks

**DOI:** 10.1038/s41598-024-56636-8

**Published:** 2024-04-09

**Authors:** WeeLiam Khor, Yichen Kelly Chen, Michael Roberts, Francesco Ciampa

**Affiliations:** 1 Department of Mechanical Engineering Sciences, University of Surrey, Guildford, GU2 7XH UK; 2 Department of Technology, Design and Environment, Oxford Brookes University, Wheatley, OX33 1HX UK; 3 Department of Applied Mathematics and Theoretical Physics, University of Cambridge, Wilberforce Road, Cambridge, CB3 0WA UK; 4 Department of Medicine, University of Cambridge, Hills Road, Cambridge, CB2 2QQ UK

**Keywords:** Mechanical engineering, Techniques and instrumentation

## Abstract

This paper presents a study on the effectiveness of a convolutional neural network (CNN) in classifying infrared images for security scanning. Infrared thermography was explored as a non-invasive security scanner for stand-off and walk-through concealed object detection. Heat generated by human subjects radiates off the clothing surface, allowing detection by an infrared camera. However, infrared lacks in penetration capability compared to longer electromagnetic waves, leading to less obvious visuals on the clothing surface. ResNet-50 was used as the CNN model to automate the classification process of thermal images. The ImageNet database was used to pre-train the model, which was further fine-tuned using infrared images obtained from experiments. Four image pre-processing approaches were explored, i.e., raw infrared image, subject cropped region-of-interest (ROI) image, K-means, and Fuzzy-c clustered images. All these approaches were evaluated using the receiver operating characteristic curve on an internal holdout set, with an area-under-the-curve of 0.8923, 0.9256, 0.9485, and 0.9669 for the raw image, ROI cropped, K-means, and Fuzzy-c models, respectively. The CNN models trained using various image pre-processing approaches suggest that the prediction performance can be improved by the removal of non-decision relevant information and the visual highlighting of features.

## Introduction

The detection of concealed weapons and improvised explosive devices by terrorists has been a constant challenge for security operations. In populated venues such as airports and football stadia, it would not be practical for the security personnel to perform manual checks on all visitors. Ideally, a stand-off and walk-through scanner would determine if a threat exists, followed by the execution of a threat isolating or removal procedure, eliminating or minimizing the harm inflicted to civilians^1^. Electromagnetic waves (EM waves) have often been exploited for non-contact object detection in human subjects^1–4^. Some of the established EM waves used in security scanning include X-ray, millimetre-waves (MMW) and terahertz^1^ (THz).

One of classic scanners is the X-ray detector^5^, which emits X-rays that are short in wavelength (10^–7^ to 10^–9^ m), high in frequency (3 × 10^16^ to 3 × 10^19^ Hz) and energy (124 keV to 145 eV). X-rays are the most penetrative among commercial EM scanners, giving full information on the cross section of the subject. However, the use of X-ray scanners is gradually being phased out due to the potential health implications when human subjects are exposed to ionizing radiation^6–10^. On the other hand, longer wavelength EM waves, such as MMW and THz have been effective in personnel security scanning applications. MMW has a frequency between 30 and 300 GHz with a wavelength between 1 and 10 mm, and can penetrate low visibility conditions, such as fog, to reflect off the subject^11–13^. The penetration capability of MMW have led to the development of a commercial personnel threat detection system by QinetiQ, the SPO-NX^14^. Like MMW, THz enables good penetration through general clothing^15,16^. THz has a frequency span between 100 GHz and 10 THz, and a wavelength between 3 mm and 30 μm. The penetrative capability of THz has led to the development of a commercial personnel scanning system by Thruvision^17–19^. Infrared (IR), when compared to MMW and terahertz, has a shorter wavelength (760 nm to 50 μm) and higher frequency (up to 214 THz). IR thermography captures thermal radiation emitted by objects in the form of image. There are two distinct forms of thermography: active thermography, where an IR illuminator (e.g., a flash or heater) illuminates the surface of the object, and passive thermography, where the IR radiation emitted from the surface of the object is directly sensed by the IR camera. Based on the black body radiation law, all objects above absolute zero degrees emit IR radiation, and variations of the radiation intensity are correlated to temperature gradients.

The potential use of IR technology in security scanning has been explored with various level of success^3,20–27^. In cases of stand-off scanning for concealed object detection, the human subject acts as the heat source for passive emission sensing. The heat emitted from the body is absorbed by the concealed object and transferred to the clothing before emission off the surface of the top clothing layer. This generally results in a temperature gradient between the concealed object area and the clothing surface. However, emission signals detected from IR systems are less distinctive compared to the MMW and THz counterpart, as the wave penetration through clothing increases with wavelength^28^. As a result, a low signal is expected when scanning through thicker layers of clothing using IR thermography^21,29,30^. Despite these physical limitations, there are also inherent advantages of using IR systems for security scanning. IR has a shorter wavelength than MMW, which means it is possible to have a higher resolution when constructed as an image for visualisation, allowing detection from longer distances^4,20,31^. It also enables higher-volume applications since multiple subjects can be visualised with larger fields-of-view. Additionally, images of faces captured using passive thermography are difficult to recognise, thus providing an additional layer of privacy^23^.

In the context of concealed object scanning, most works have resorted to MMW or THz since they enable better penetration. Even though IR was less explored in this aspect, machine learning (ML)-based techniques have been successfully implemented with IR data, e.g., for non-destructive damage inspection and segmentation of moving objects^32–37^. In an ideal concealed object detection system, the decision made upon scanning of the subject should be decisive and accurate. Due to the penetration limitation of IR systems, it could be difficult for an operator to make consistent decisions, particularly in cases of layered clothing.

The proposed approach in this paper to address this issue is by implementing convolutional neural networks (CNN) for concealed object detection and classification. Utilizing the transfer learning approach, a pre-trained CNN was fine-tuned using application-specific data, i.e., IR images of subjects with and without concealed objects. Transfer learning has been successfully implemented in many areas, such as biomedical imaging^38–42^, non-destructive testing in buildings^43^, agricultural pest and disease management^44,45^, topology segmentation^46^, image enhancement^47^, pedestrian identification^48^, and security scanning^5,28^. The aim of this work is to explore the potential of using CNNs to predict the presence of a target object concealed underneath layered clothing using IR thermography. Raw IR images and pre-processed images were used to fine-tune a pre-trained ResNet-50 CNN model. This was followed by an evaluation of model performance using receiver operating characteristic (ROC) curves.

## Methodology

### Data acquisition

**Figure 1:**
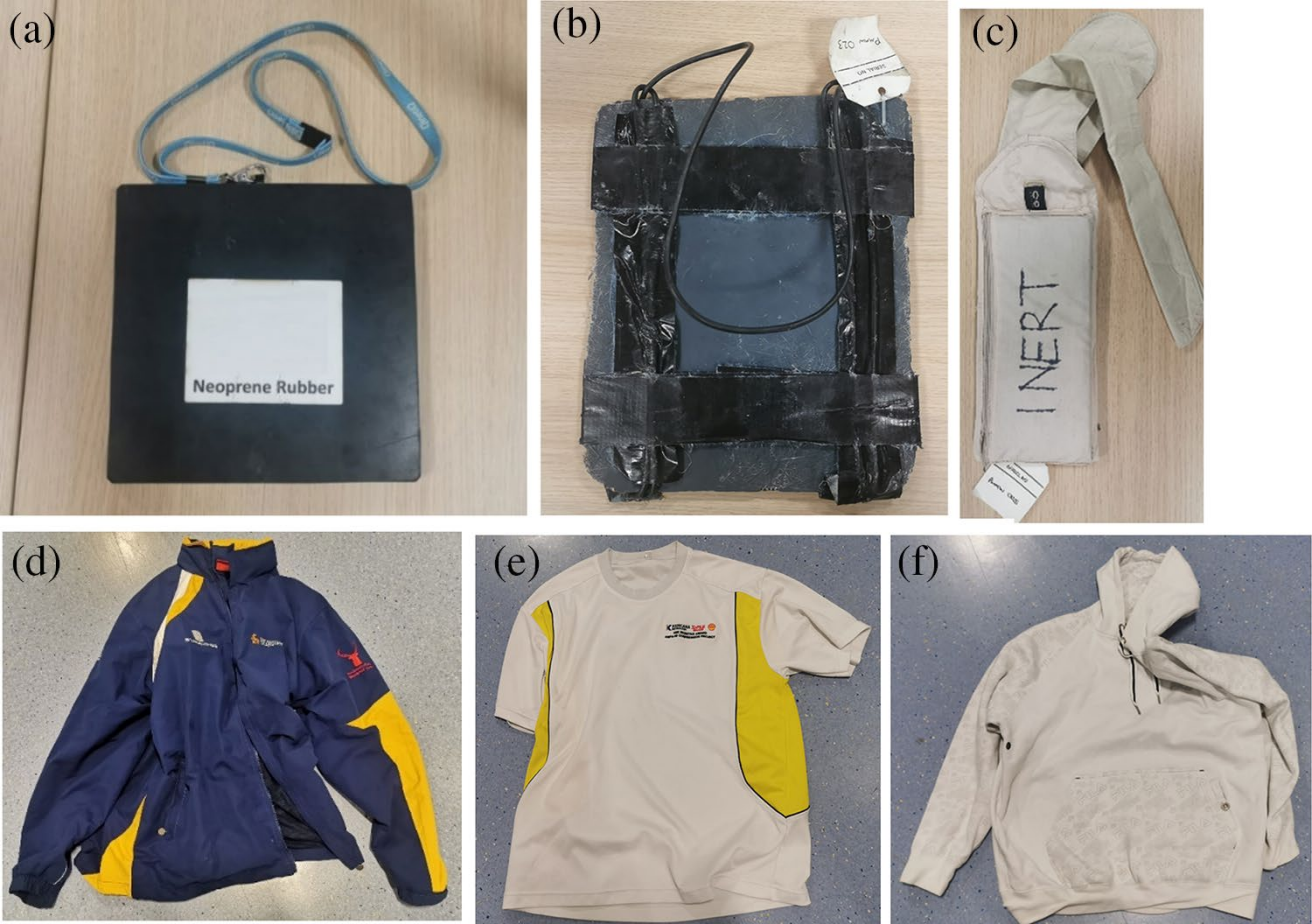
Photographs of simulated concealed weapons including (**a**) neoprene rubber block, (**b**) metal bearings in clay, and (**c**) fragments in money bag. Examples of outer clothing such as (**d**) layered windproof, (**e**) t-shirt, and (**f**) hoodie.

A FLIR A6750sc Mid Wavelength IR camera with a 3–5 µm waveband, < 20 mK thermal sensitivity (NETD), and a focal plane array size of 640 × 512 pixels was used to capture thermal data. Examples of clothing and simulated concealed weapons used in data collection are shown in Fig. 1.

### Image pre-processing

Four different pre-processing methods were investigated in this work: raw data from the IR camera, cropped region-of-interest (ROI), K-means clustered, and Fuzzy-c clustered images. This enabled us to investigate the effects of background removal, clustering, and information reduction on the trained CNN model for classification.

The ROI images were produced by cropping the subject from the raw image. Based on the generalised assumption of a 32 °C skin temperature, a threshold of 30 °C was used to crop the subject. This was applied on the raw image, where the first and last time the threshold temperature was encountered when reading temperature values from left to right of the image, and the first time when reading from top to bottom. This retains some of the exposed skin of the subject within the ROI image, removing some background information.

The K-means^49^ and Fuzzy-c^32^ methods are unsupervised ML techniques used for information reduction. K-means is a well-known clustering method whereas Fuzzy-c is a less famous variation. Both approaches commence by establishing cluster centres to initiate the cluster affiliation. This is followed by computing membership scores based on distances between datapoints (pixels) and the cluster centres, which reveal the degree of association between each pixel and each cluster. These scores guide the adjustment of cluster centres, with more weights placed on pixels that exhibit stronger association. This iterative procedure continues until a defined stopping criterion is satisfied. The application of such information reduction technique on images will typically simplify the illustration of images. The primary distinction between these two methods lies in the fact that K-means employs hard clustering, restricting each data point to a singular cluster association (usually the nearest cluster centre)^49^. Conversely, Fuzzy-c utilises a soft clustering approach, enabling each data point to be linked with all available clusters while assigning distinct degrees of importance (or weight)^50^. The weighting (or membership score) is computed as,

1 $${\mu }_{ij}=\frac{1}{{\sum }_{k=1}^{C}{\left(\frac{{D}_{ij}}{{D}_{kj}}\right)}^{\frac{2}{{\text{m}}-1}}}, 1\le {\text{i}}\le C, 1\le j\le {\text{N}},$$

where *μ*_*ij*_ is the membership score of the *j*-th pixel (N pixels in total) for the *i*-th cluster (C clusters in total); *D*_*ij*_ is the distance (i.e., intensity difference) between the *j*-th pixel and the centre of the *i*-th cluster; and m is a parameter to control the extent of the fuzzy overlap. Updated cluster centres are subsequently computed as,

2 $${c}_{i}=\frac{{\sum }_{j=1}^{N}{\mu }_{ij}^{m}{x}_{j}}{{\sum }_{j=1}^{N}{\mu }_{ij}^{m}}, 1\le i\le C,$$

where *c*_*i*_ is the updated centre of the *i*-th cluster and *x*_*j*_ is the intensity of the *j*-th pixel. Furthermore, there is a disparity in the stopping criteria between K-means and Fuzzy-c. In K-means, the algorithm halts when cluster centres remain constant between consecutive iterations. In contrast, for Fuzzy-c, the algorithm continues its iterations until there is no further enhancement in the objective function. This objective function *J* is defined as

3 $$J={\sum }_{{\text{i}}=1}^{{\text{C}}}{\sum }_{{\text{j}}=1}^{{\text{N}}}{\mu }_{{\text{ij}}}^{{\text{m}}}{D}_{{\text{ij}}}^{2}$$

On general consensus, Fuzzy-c is considered more robust compared to K-means^51^. However, this could differ depending on application scenarios^52^. The thermal intensity data (apparent temperature) obtained from the IR camera was flattened into 1D before the pixel intensities enter the clustering algorithm. This allows the clustering algorithms to cluster the data relative to the apparent temperature in 1D, instead of area clustering in 2D. A total of 16 cluster centres were set to illustrate a total of 16 intensity bins in each image. Commercial numerical computing software, MATLAB^53^, was used to process images and train models.

### Convolutional neural networks, CNN

**Figure 2:**
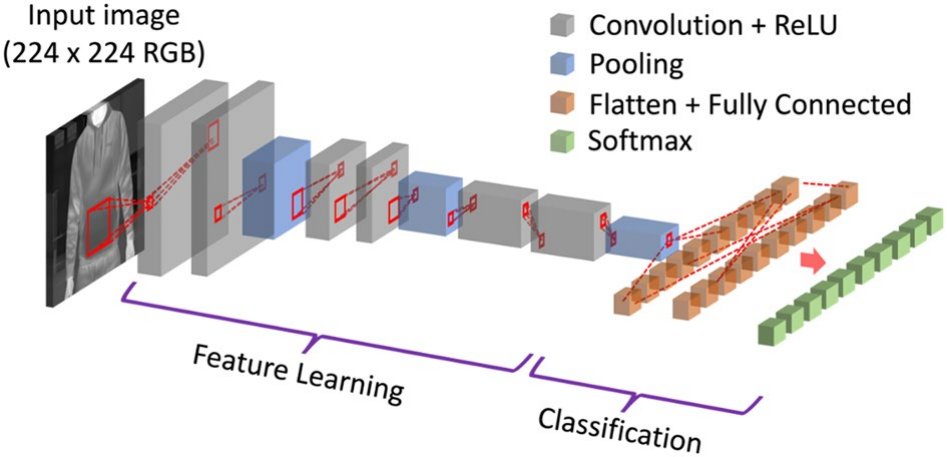
Architecture of the CNN image classification-based framework with multiple convolutional layers.

A ResNet-50 CNN model^54^ was used to classify IR images. CNNs are a class of deep learning network architectures that can be applied to the classification of audio, time-series, signals, and most commonly for image classification. In the case of image classification, the input image is passed through a set of convolutional filters, each of which extract certain features from the images, e.g., brightness and edges Fig. 2.

The rectified linear unit (ReLU) serves as an activation function for the convolutional filter, and the pooling layer performs linear downsizing on the output, reducing the number of parameters that the network needs to learn. In the classification layers, the data is flattened into 1D before passing through a classification neural network, typically consisting of a fully connected network (also described as dense layer) and a SoftMax activation which normalises the output into probabilities. Typically, to train a CNN model from scratch, the general consensus requires that the size of the training dataset be at least one magnitude higher than the size of the test dataset to improve diversity and avoid overfitting^55,56^. However, well-labelled IR datasets for subjects with concealed objects are rare and often not publicly shared. To tackle the small-dataset problem, a pre-trained ResNet-50 model was used. The model was pre-trained using the ImageNet dataset^57^, which contained 1.4 million natural images with 1000 classes. In the transfer learning step, the fully connected layer was fine-tuned using a total of 900 labelled images (462 with and 438 without object). The underlying assumption in the transfer learning approach is that generic features extracted from an exceptionally large dataset are also present and informative in different datasets. This portability of learned generic features is a unique advantage of deep learning that makes itself useful in various domain tasks with small datasets^58^. Using MATLAB’s Deep Network Designer, the fully connected layer in the pre-trained model was modified to train using a weight learn rate factor and bias learn rate factor of 10. During training, argumentations were applied to the training images to improve the diversity of data and to avoid overfitting. Transformations were performed by applying reflection on the Y-axis, rotation by ± 45°, and rescaling by ± 25%. Internal validation was performed using 30% of randomly sampled training data to monitor the training process. Stochastic gradient descent was used as the optimiser with an initial learning rate of 0.0001, validation frequency of 5, maximum epoch of 20 and a mini batch size of 10. Validation accuracies of 98.95%, 97.19%, 94.07%, and 95.19% were achieved for the raw image, ROI, K-means, and Fuzzy C image models, respectively.

**Figure 3:**
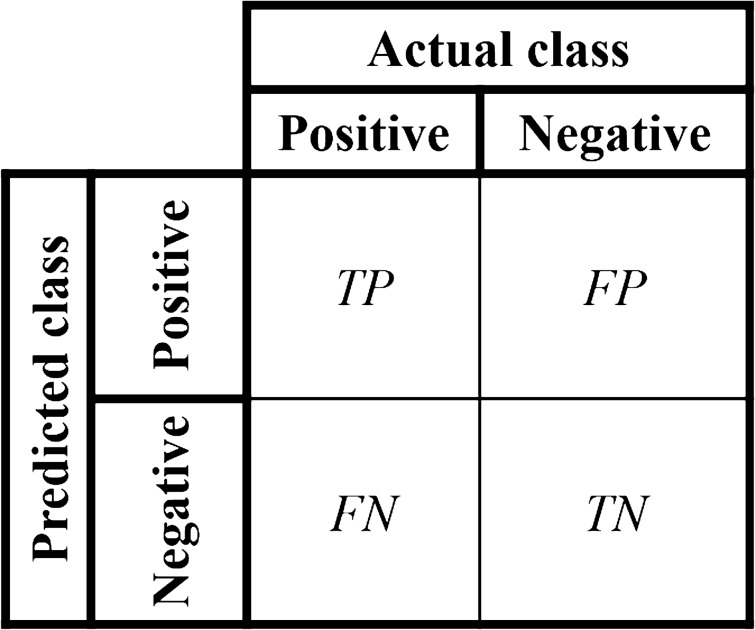
Confusion matrix for model evaluation.

After training, a total of 200 images (equal split with and without object) that have not been exposed to the models (internal holdout/test-set) were pre-processed into the respective image type to evaluate the fine-tuned ResNet-50 models. When a cut-off is applied to the predicted probabilities, the comparison of the predicted and the true label will result in a confusion matrix consisting of true positives, false positives, false negatives, and true negatives Fig. 3. True positives and true negatives correspond to correct predictions, whilst false positives and false negatives are wrong predictions.

To evaluate the model performances without assuming a cut-off on the predicted probabilities, a receiver-operator characteristic (ROC) curve was used. This is a 2D diagram that is built using the true positive rate (TPR, also described as sensitivity or recall) and false positive rate (FPR, also described as false alarm rate) calculated using results obtained from the confusion matrix using each predicted probability as a tentative cut-off. These parameters are given by,

4a $$TPR=\frac{\Sigma TP}{\Sigma \left(TP+FN\right)},$$

4b $$FPR=\frac{\Sigma FP}{\Sigma \left(FP+TN\right)}.$$

**Figure 4:**
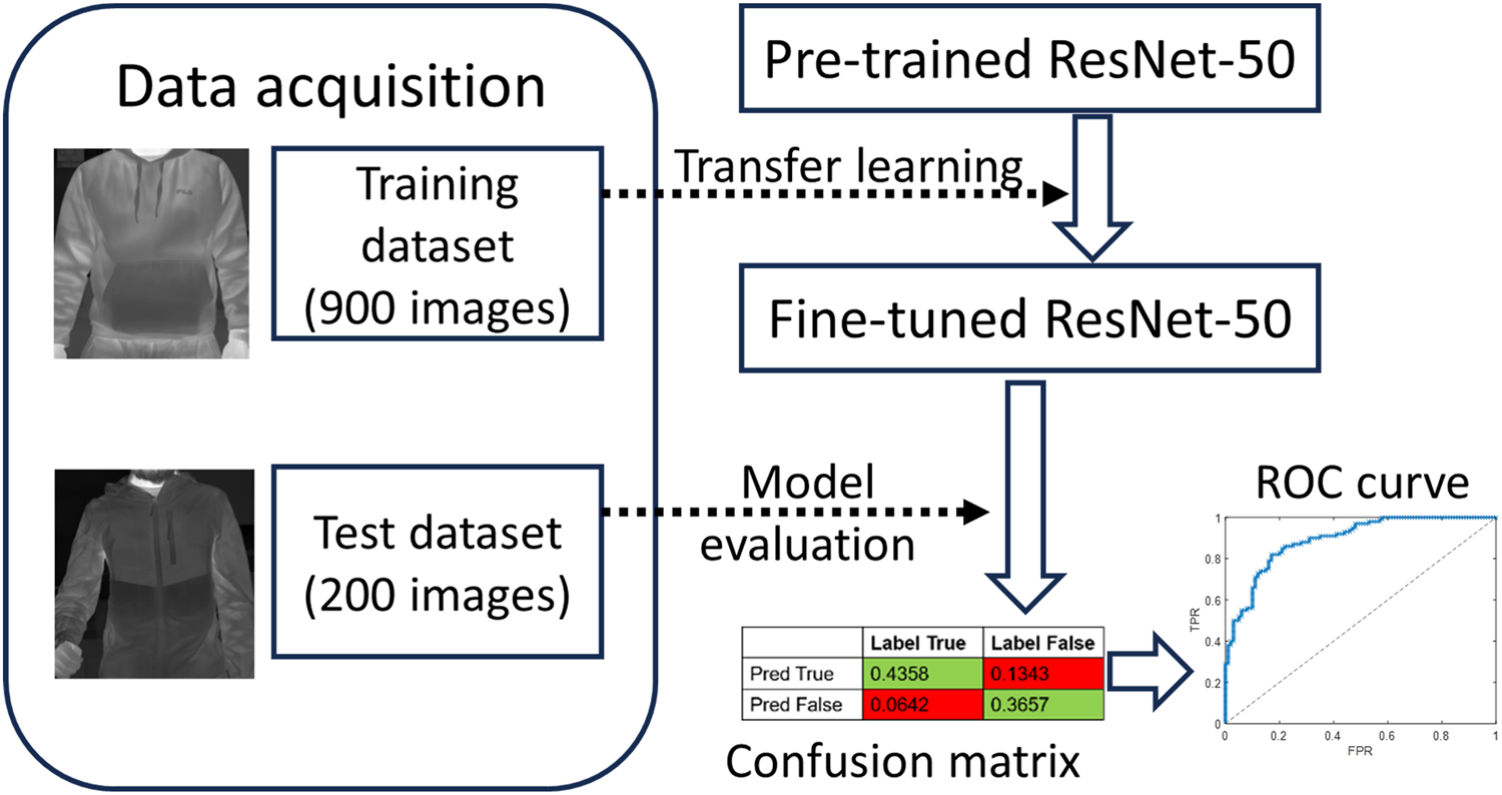
An illustration of the training and evaluation process of the ResNet-50 models for each of the different image type.

The TPR and FPR are metrics to evaluate the correct predictions of the model. Integrating the ROC curve, an area-under curve (AUC) can be obtained. The AUC provides a performance metric for the models, where 0.5 suggests no discrimination, 0.7–0.8 is considered acceptable, 0.8–0.9 is considered excellent, and > 0.9 is outstanding^59^. The diagram in Fig. 4 illustrates the process of training and evaluation of the ResNet-50 models.

## Results and discussion

**Figure 5:**
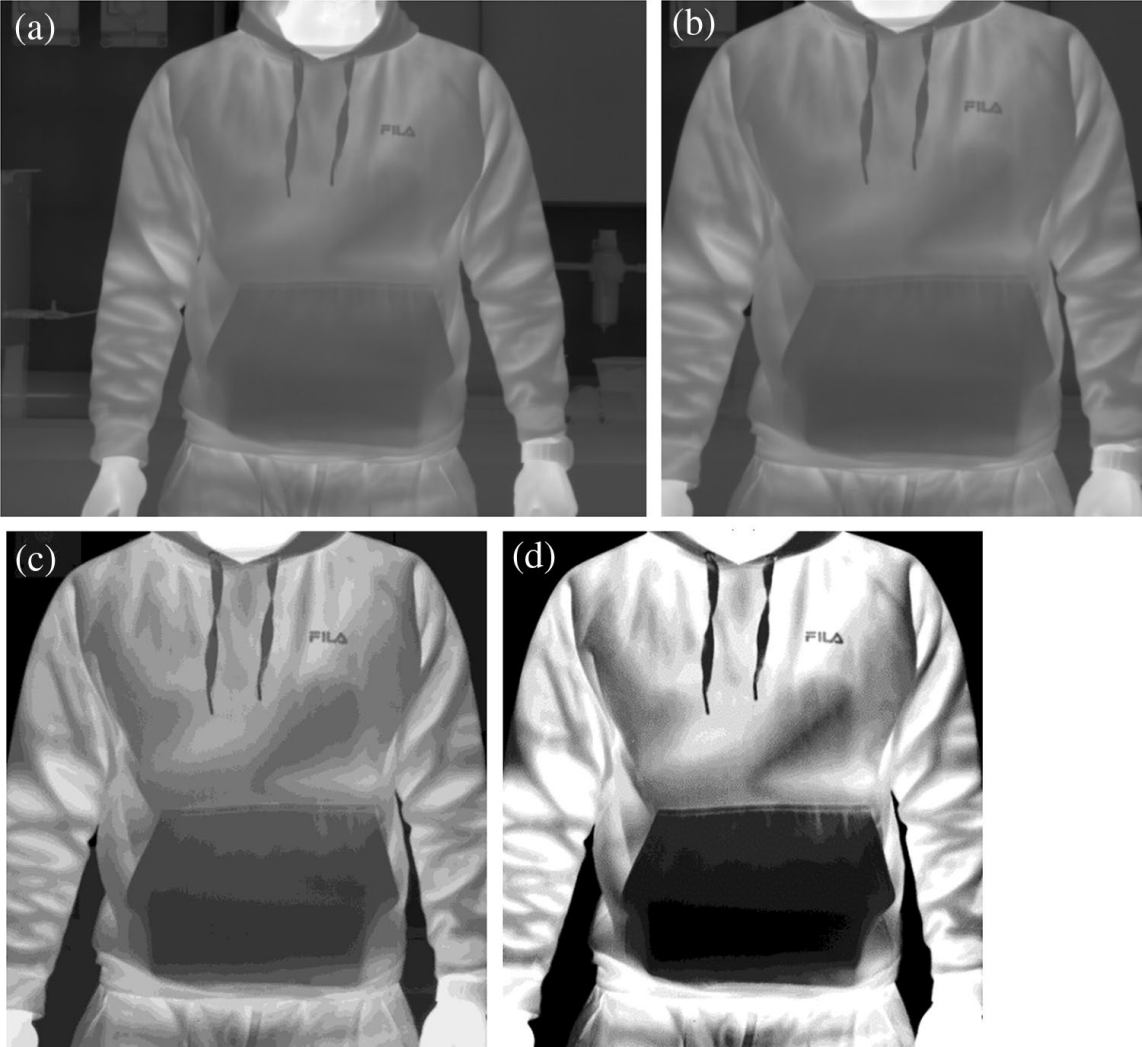
An example of the dataset image with a hidden rectangular box in the pocket, showing the (**a**) raw image, (**b**) ROI sectioned image, (**c**) K-means clustered image, and (**d**) Fuzzy-c clustered image.

The raw image illustrates the thermal intensity data captured by the camera data in 256 intensity values Fig. 5a. The ROI image crops the subject using a rectangular bounding box, removing excess background from the image Fig. 5b. An illustration of the K-means and Fuzzy-c post-processed images are shown in Fig. 5c,d respectively. The K-means and Fuzzy-c images looked like the ROI image, but with reduced information, as the images were illustrated with 16 grayscale bins instead of 256 bins. The cluster centre distribution in the K-means image is more even, making it look more similar to the ROI image. In contrast, in the Fuzzy-c image, clusters are more 'polar,' with distinct separation between cold and warmer areas.

**Figure 6:**
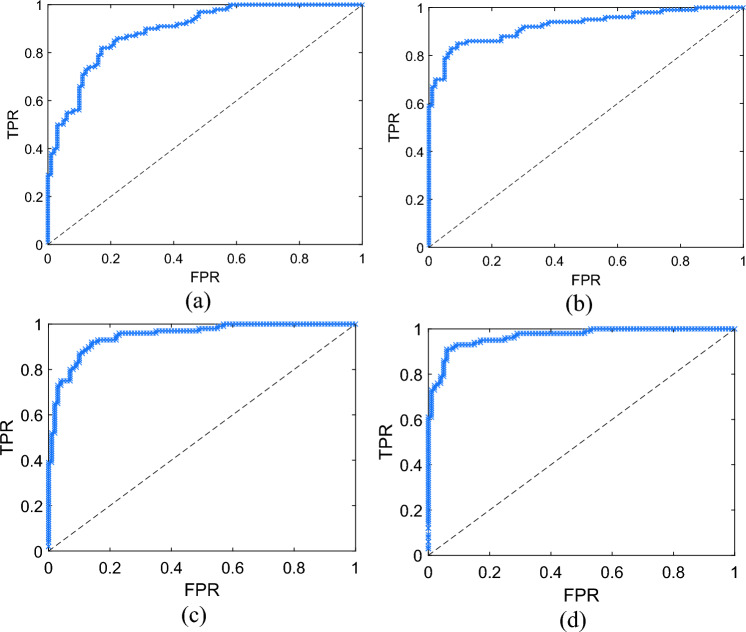
Receiver-operator Characteristics curves for the model trained using (**a**) raw images, and (**b**) ROI cropped images, (**c**) K-means clustered, and (**d**) Fuzzy-c clustered images.

The test-set ROC curves obtained from each model are shown in Fig. 6, revealing an AUC of 0.8923, 0.9256, 0.9485, and 0.9669 for the raw image model, ROI sectioned image, K-means and Fuzzy-c clustered image model, respectively. Based on AUCs, it is clear that pre-processed images tend to perform better than unprocessed data. Subject isolation by ROI cropping performed better than the raw image model, where some background not indicating the object presence was removed. K-means and Fuzzy-c both resulted in an improved background removal, where the background was entirely represented by a cluster. This offers a potential explanation to their superior model performance compared to the ROI cropped model. In the K-means model, the clustered image seemed more like the raw data where cluster transition is more gradual. In Fuzzy-c, the clustered image was more ‘polar’, where the clothing showed a brighter cluster, and the object area was significantly darker. This could be a reasoning to the improved performance in the Fuzzy-c model compared to the K-means model, where the object area is more ‘obvious’, allowing the CNN model to have more distinct features during the training process. Considering the improved performance of the post-processed image models, it is hypothesised that 2D clustering (1D clustering was used above), or advanced segmentation techniques could potentially further improve the performance of CNN models.

In the current study, concealed objects exhibited lower temperatures compared to the subjects. Consequently, pockets of lower apparent temperature, detected by the IR camera, indicated the presence of concealed objects on the surface of the clothing. Most of the clothing types used in this practice were mono-material, where the exposed clothing surface exhibited similar emissivity. However, in complex clothing, such as those comprising two or more materials with distinctly different emissivity, pockets or regions of low temperature can be 'artificially' created.

**Figure 7:**
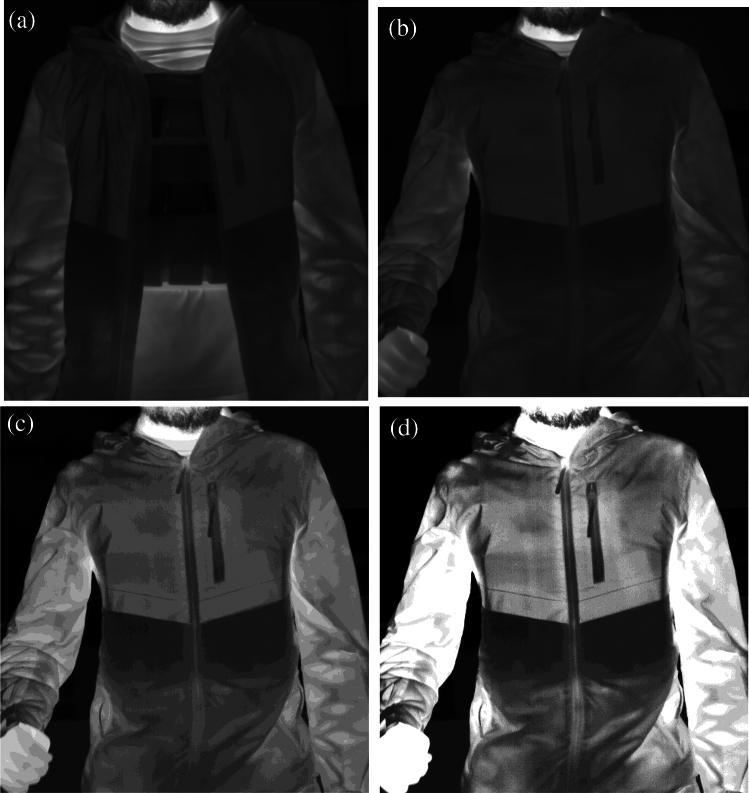
An example of a complex clothing with dissimilar materials with different emissivity. The ROI sectioned image with exposed object shown in (**a**), whilst the concealed object images were shown in (**b**) ROI sectioned, (**c**) K-means clustered, and (**d**) Fuzzy-c clustered.

An image with an object concealed under complex clothing material is shown in Fig. 7. In the concealed object area, the torso area above the elbow exhibited a higher temperature reading compared to the area directly below the elbow, with a distinct border corresponding to the clothing design. The top part of the jacket is made of polyester (PET) with an approximate emissivity of 0.80, while the lower region of the jacket is made of cotton, with an emissivity of ~ 0.67^60^. Therefore, the development of a robust model with the ability to identify concealed objects in complex clothing requires a large dataset with a variety of complex clothing materials for training.

## Conclusions

Convolutional neural network, ResNet-50 was used to classify infrared images of subjects with concealed objects under clothing. The transfer learning approach was used to fine tune an ImageNet pre-trained model. Four models were developed and evaluated: a raw image model, ROI cropped model, K-means model, and Fuzzy-c model, which produced ascending improvement in model performance, exhibiting area-under-curve of 0.8923, 0.9256, 0.9485, and 0.9669, respectively. Pre-processing image data by removing non-decision relevant information from the images, such as background information, and visual highlighting of the object area effectively resulted in automated and enhanced model prediction performance. Regardless of image processing techniques, the application of transfer learning on a robust pre-trained network was shown to be effective on such small dataset problem.

## Data Availability

The data that support the findings of this study are available from the corresponding author, but restrictions apply to the availability of these data, which were used under license for the current study, and so are not publicly available. Data are however available from the authors upon reasonable request and with permission of the Defence Science and Technology Laboratory (DSTL), via the Defence and Security Accelerator (DASA).
